# Identification and validation of MicroRNA-mRNA Networks in Dorsal Root Ganglia after Peripheral Nerve Injury

**DOI:** 10.7150/ijms.73113

**Published:** 2022-07-11

**Authors:** Xinyi Gu, Hao Guo, Canjun Zeng, Yijun Liu

**Affiliations:** 1 Department of Orthopedics and Trauma, Peking University People's Hospital, Beijing, China, 100044.; 2 Department of Foot and Ankle Surgery, Center for Orthopaedic Surgery, the Third Affiliated Hospital of Southern Medical University, Guangzhou, China.; 3 Orthopaedic Hospital of Guangdong Province, Guangzhou, China.

**Keywords:** transcriptome, mRNA, microRNA, transcription factors, peripheral nerve injury

## Abstract

Changes in DRG after nerve injury involve neuronal damage, apoptosis, pain transmission, and activation of regenerative programs. It is unclear which genes and microRNAs may play a major role in this process. Therefore, this study performed a meta-analysis of previously published gene expression data to reveal the potential microRNA-mRNA network in dorsal root ganglia (DRG) after peripheral nerve injury. We searched 5 mRNA and 3 microRNA expression data sets, obtained 447 differentially expressed genes (DEGs) and 5 differentially expressed miRNAs, determined the biological pathways enriched by these DEGs, and further predicted new microRNA-mRNA interactions, such as miR-21/Hmg20a, miR-221/Ube2ql1, miR-30c-1/Rhoq, miR-500/Sema3c, and miR-551b/Cdc42se2. We verified these hub mRNA and miRNA in rats by qRT-PCR and found the results were consistent with the bioinformatics analysis. And we predicted transcription factors associated with these genes (gTFs) and TFs associated with these microRNAs (mTFs) and constructed the mTF-miRNA-gene-gTF regulatory network to further explore the molecular mechanism in DRG. Finally, we compared the DRG transcriptome after PNI to that of chronic constriction injury (CCI), and found that PNI caused greater damage to DRG compared to CCI. At the same time, the related mechanisms of pain caused by the two pathophysiological process may be different.

## Introduction

DRG plays an important role in the communication between the peripheral and central nervous systems, which is also the primary afferent neuron to produce nociception 1. Peripheral nerve injury can lead to damage of DRG neurons and also the connections between neurons, resulting in abnormal sensory conduction and neuropathic pain. Gene expression in DRG is dynamic and can respond to damage 2. When stimulated by injury, the phenotype and the functional state of DRG will undergo significant changes, which make DRG become the origin site of pain signal transmission 3. At the same time, axon injury of peripheral DRG neurons will initiate the regeneration process, and ultimately promote the regeneration of axons 4, 5. The regenerative ability of DRG neurons after injury is called the modulated injury effect. Conditioned injury induces regeneration by increasing neuronal regeneration capacity. In order to achieve this, activation of the damage signal response is crucial 6, 7. To explore the transcriptome changes of DRG after peripheral nerve injury will help us to have a more comprehensive understanding of the role of DRG in peripheral nerve injury, especially the role in pain transduction and axon regeneration.

Molecules of DRG related to pain transduction mainly include Purinergic P2X Receptors (P2Xs), Transient Receptor Potential Receptors, Voltage-Gated Sodium Channels (NaVs) and Voltage-Gated Calcium Channels(CaVs) 8. P2X receptor was expressed in spinal cord, dorsal horn and DRG neurons 9, 10. Studies have shown that the isopurinergic receptors P2X3 and isopurinergic receptors p2X2/3 play important roles in spinal cord nociceptive transmission 11. Therefore, P2X2/3 has been proposed as a potential therapeutic target for peripheral neuropathic pain. TRP receptors are divided into several subfamilies, among which heat-sensitive TRP channel-related proteins TRPV, TRPA, and TRPM in DRG are closely related to pain transmission^12^-^16^. DRG neurons have five NaV subtypes 17. Hyperexcitation of neurons after peripheral nerve injury may result from changes in the activity of voltage-gated sodium channels, which results in pain or hyperalgesia 18, T-type CaV, CaV3.2 and CaV3.3, are expressed in DRG neurons and have shown potential as therapeutic targets for neuropathic pain 19, 20. Given the complexity of DRG phenotype and functional changes following injury, the identification of key factors and signaling pathways based on global analysis may help to understand the underlying molecular mechanisms of neuropathic pain.

The DRG response after peripheral nerve injury is beneficial to axon regeneration. The proteins activated in neuron regeneration mainly include growth associated protein 43 (GAP43), cortical cytoskeleton associated protein 23 (CAP23), superior cervical ganglion 10 (SCG10), or small proline-rich repeat protein 1A (SPRR1A) 21. During this process, cytoskeletal components, cytoplasmic proteins, vesicles and organelles of multiple structures need to be transported to the axon terminals to play their roles. Although there is evidence that axon transport plays a central role in regulating internal axon regeneration, the regulation of transport by injury remains unclear 22. Analysis of the DRG transcriptome will help us understand this regulation.

MicroRNAs are a class of non-coding RNAs that can regulate gene expression 23, which have shown promising prospects as potential therapeutic targets for neuropathic pain and axonal regeneration 24-27. Studies have shown that reduction of miR-96 expression in DRG by drugs could improve Nav1.3 expression and alleviate mechanical and thermal hyperalgesia 28. Mir-155-5p was shown to promote DRG neuronal axon growth through the cAMP/PKA pathway 27. Thus, the identification of microRNAs could broaden the knowledge of global gene expression regulation in DRG after nerve injury.

We screened and systematically integrated mRNA and microRNA expression profiles of DRG after PNI to capture the most relevant microRNA-mRNA regulatory network. Our analysis also identified molecular pathways that may be highly involved in DRG phenotypic changes after PNI. At the same time, we compared the DRG transcriptome after PNI with that after CCI. CCI is a classical model for neuropathic pain, which has little damage to nerves. Such comparison will help us better understand the characteristics and mechanism of DRG in peripheral nerve injury 29.

## Materials and Methods

### Inclusion criteria for datasets

NCBI-GEO (http://www.ncbi.nlm.nih.gov/geo/) and PubMed (http://www.ncbi.nlm.nih.gov/pubmed) were used for the dataset retrieval. The meta-analysis was conducted following the PRISMA Statement 30.

The keywords used for gene expression data were: “DRG AND Nerve Injury”, “dorsal root ganglia AND Nerve Injury”, and “DRG AND Nerve Injury AND sequencing”. These meta-analysis searches comprised studies published between 2010 and 2021. Our inclusion criteria were (1) gene expression data in DRG with peripheral nerve injury, (2) the number of samples in each group should be greater than two, (3) the duration of nerve injury was within one month, (4) all types of peripheral nerve injury were considered, (5) the inclusion of normal tissues for comparison, and (6) all gene expression analysis platforms were considered. Our exclusion criteria were (1) non-DRG samples, (2) chronic constriction injury, (3) non-mRNA datasets, and (4) review studies.

The keywords used for microRNA expression data were: “DRG AND Nerve Injury”, “dorsal root ganglia AND Nerve Injury”, and “DRG AND Nerve Injury AND sequencing”. These meta-analysis searches comprised studies published between 2010 and 2021. Our inclusion criteria were (1) microRNA expression data in DRG with peripheral nerve injury, (2) the number of samples in each group should be greater than two, (3) the duration of nerve injury was within one month, (4) all types of peripheral nerve injury were considered, (5) the inclusion of normal tissues for comparison, and (6) all microRNA expression analysis platforms were considered. Our exclusion criteria were (1) non-DRG samples, (2) chronic constriction injury, (3) non-microRNA datasets, and (4) review studies.

In this study, edgeR packages were used for gene differential expression analysis, gene/microRNA with adj p-value < 0.05 were considered to be differentially expressed.

### Meta-analysis of global gene and microRNA datasets in DRG after PNI

Meta-analysis was performed using Vote counting generic ways of combining information^31^, and the results were presented in the form of Venn diagrams by the graphing software (ORIGIN2019; OriginLab, Northampton, MA).

### GO and KEGG Enrichment Analysis

Gene Ontology Analysis (http://www.geneontology.org) and Kyoto Encyclopedia of Genes and Genomes (KEGG) Analysis (http://www.genome.ad.jp/kegg/) were conducted for DEGs respectively. GO term/ KEGG pathway with adj p-value < 0.05 was considered to be significantly enriched. Bar chart of GO terms was drawn by Microsoft Excel 2016. KEGG network was visualized by ggplot2 packages.

### Construction of protein-protein interaction (PPI) network and module analysis

The DEGs obtained in this study were imported into the STRING v.10.5 database 32 to construct a PPI network and the threshold was set to 0.4. The PPI network was visualized by Cytoscape software v. 3.7.2 33, and cytoHubba^34^ was used to calculate the degree of each node. The degree represents the number of interactions of a specific protein. In this network, we showed the top10 genes with the highest degree.

The plugin MCODE in Cytoscape was used for module analysis, and subnets (modules) were sorted by the score after they were obtained. For genes in each module, the degree was calculated using cytoHubba and the node size was adjusted according to the degree.

### Prediction of microRNA target genes

Multiple algorithms (TargetScan 35, miRDB 36, and MicroT-CDS 37) were used to predict microRNA target genes, and then the three results were intersected to obtain the predicted target genes of each microRNA.

### Animal model establishment for verification

Specific-pathogen-free, male, Sprague-Dawley (SD) rats, aged 2 months and weighing 250-300 g, were purchased from Charles River (Boston, MA, USA). 12 rats were randomly divided into two groups (n=6 for each group): sham group and PNI group. The PNI group was anesthetized with 1.5% isoflurane (Huazhong Haiwei (Beijing) Gene Technology Co., Ltd., Beijing, China). During the operation, the sciatic nerve of the right lower limb was separated and exposed under direct vision. A 1 cm length of sciatic nerve proximal to the division of tibial and common peroneal nerves was removed. The nerve stump was ligated and reflexed to the adjacent muscle to prevent the nerve from regenerating. The sham group underwent the same surgical procedure without damaging the nerves. The protocol was approved by the Ethics Committee of the Peking University People's Hospital (Permit Number: 2020PHE089).

### Real-time quantitative PCR

The RNAprep Pure Tissue Kit (Tiangen Biotech, Beijing, China) was used to extract tissue RNA from the right L4-5 DRGs of each group. A portion of the RNA was reverse-transcribed using the 5X All-In-one RT MasterMix Kit (Abm, Vancouver, Canada) and the resulting DNA was used for quantitative analysis of the hub mRNA. Specific reverse transcription of the remaining RNA was performed using PrimeScript RT Reagent Kit (Takara, Osaka, Japan), and the resulting DNA was used for quantitative analysis of miRNA. Finally, EasyScript® first-strand cDNA Synthesis SuperMix (TransGen Biotech, Beijing, China) was used for quantitative analysis. The results were calculated by the 2^-ΔΔCt^ method 38. The primer sequences were listed as followed (Table 1).

### Construction of the mTF-miRNA-gene-gTF regulatory network

9 up-microRNA / down-gene interactions and 8 down-microRNA / up-gene interactions obtained in this study were used to construct the mTF-miRNA-gene-gTF regulatory network. The TF related to these genes was called gTF, and the TF related to these miRNAs was called mTF. The gTF information was obtained from TRANSFAC 39, and the inclusion criteria were MSS and CSS score = 1. The mTF information was obtained from TransmiR v.2.0 database 40. The mTF-miRNA-gene-gTF regulatory network was visualized by Cytoscape software v. 3.7.2 33.

## Results

### Data screening criteria

**Figure 1:**
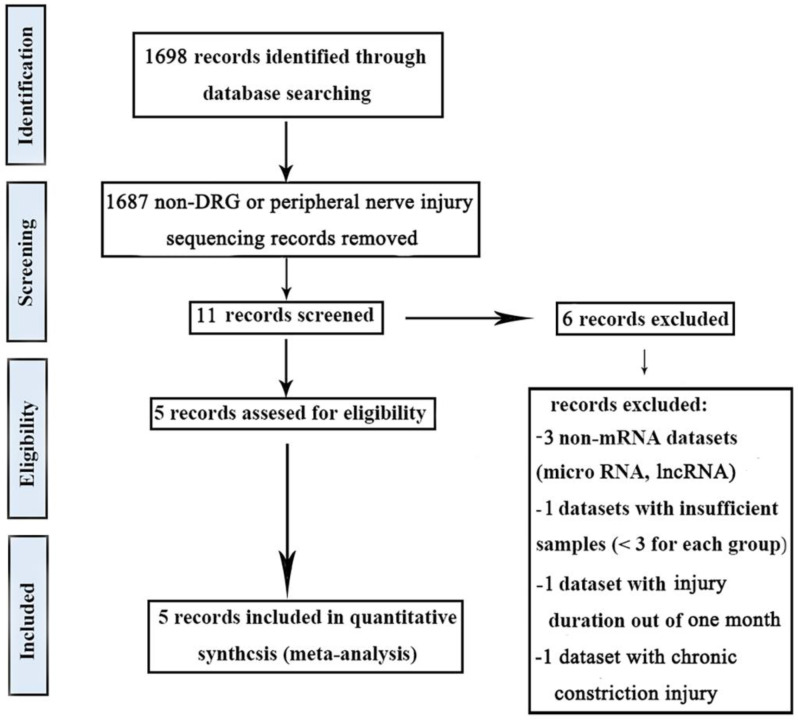
Workflow of the methodology in the meta-analysis.

In this study, five gene expression databases of DRG after PNI were included 41-43. The flow diagram of included databases was shown in Figure 1. Species sources of the five databases were mice or rats. Surgical procedures were sciatic nerve ligation or sciatic nerve resection. The sampling time was within 1 month after the operation and the sample size was not less than 3/group (Table 2).

### DEGs in DRG after PNI

**Figure 2:**
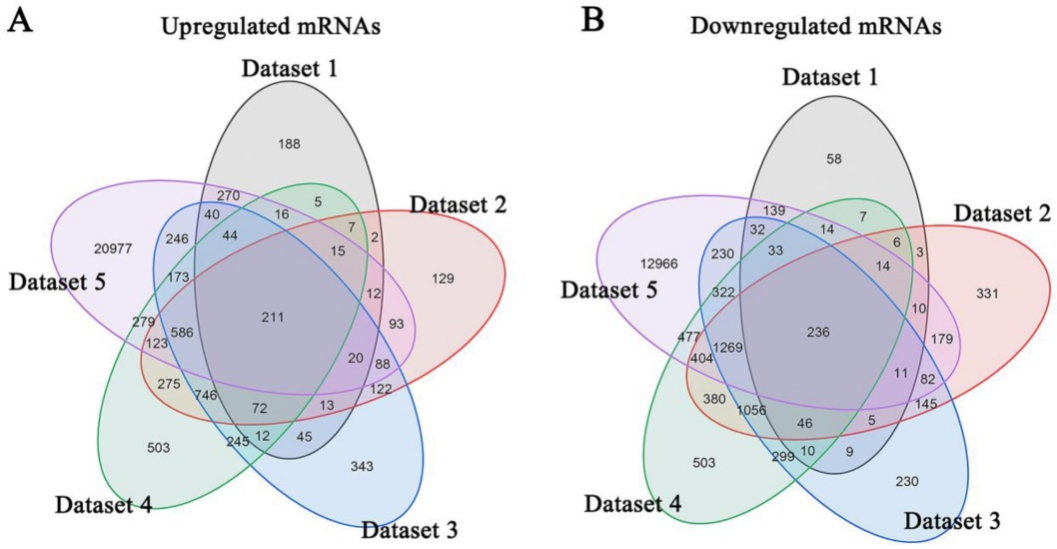
** Identification of DEGs in DRG after PNI.** (A) 211 upregulated mRNAs and (B) 236 downregulated mRNAs were identified based on 5 DRG datasets after PNI.

**Figure 3:**
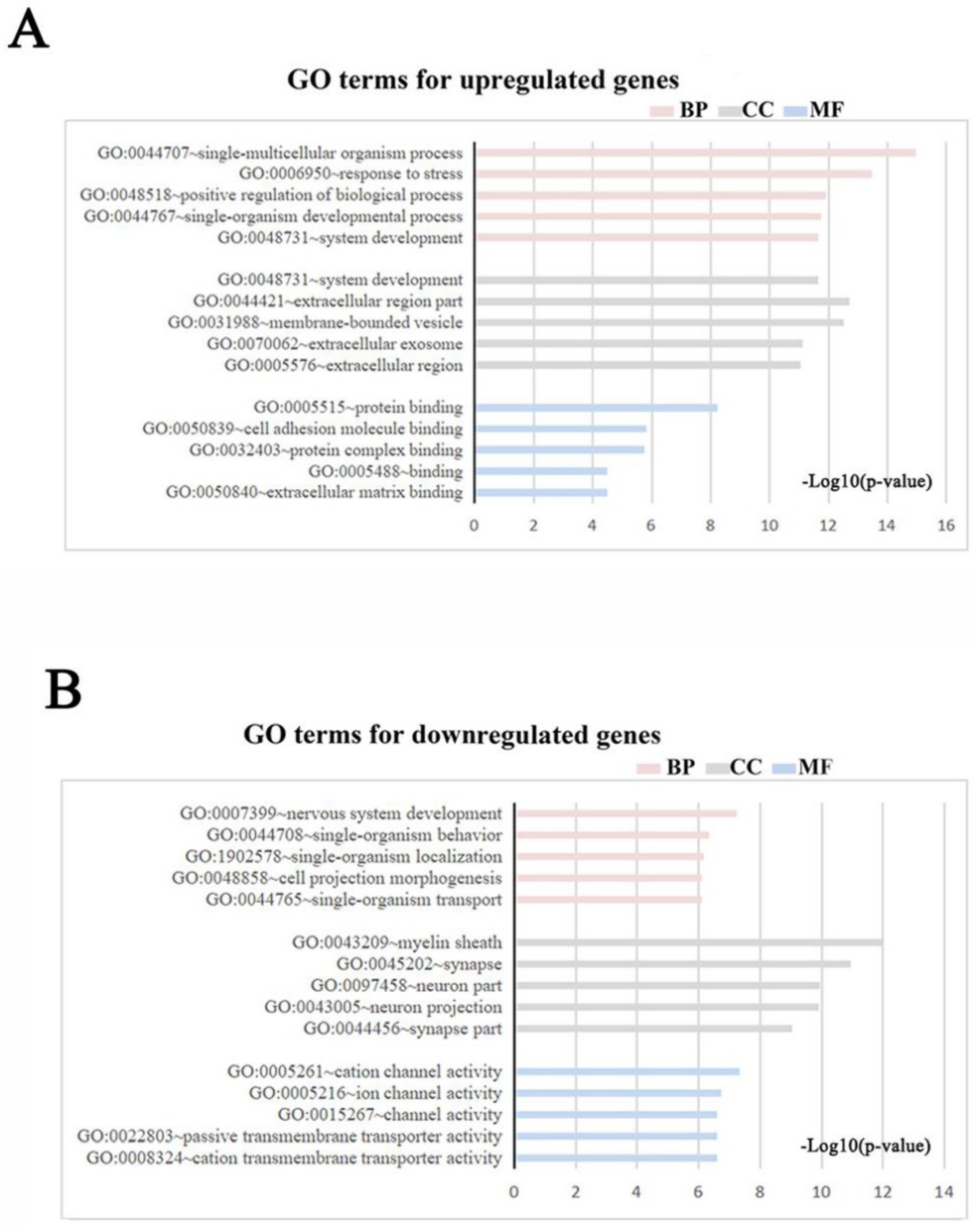
** GO terms in DRG after PNI.** (A, B) GO enrichment analyses were conducted for (A) up and (B) downregulated DEGs respectively (Each figure shows the top5 BP, CC, and MF terms with the lowest p-value). BP, biological process; CC, cellular component; MF, molecular function.

**Figure 4:**
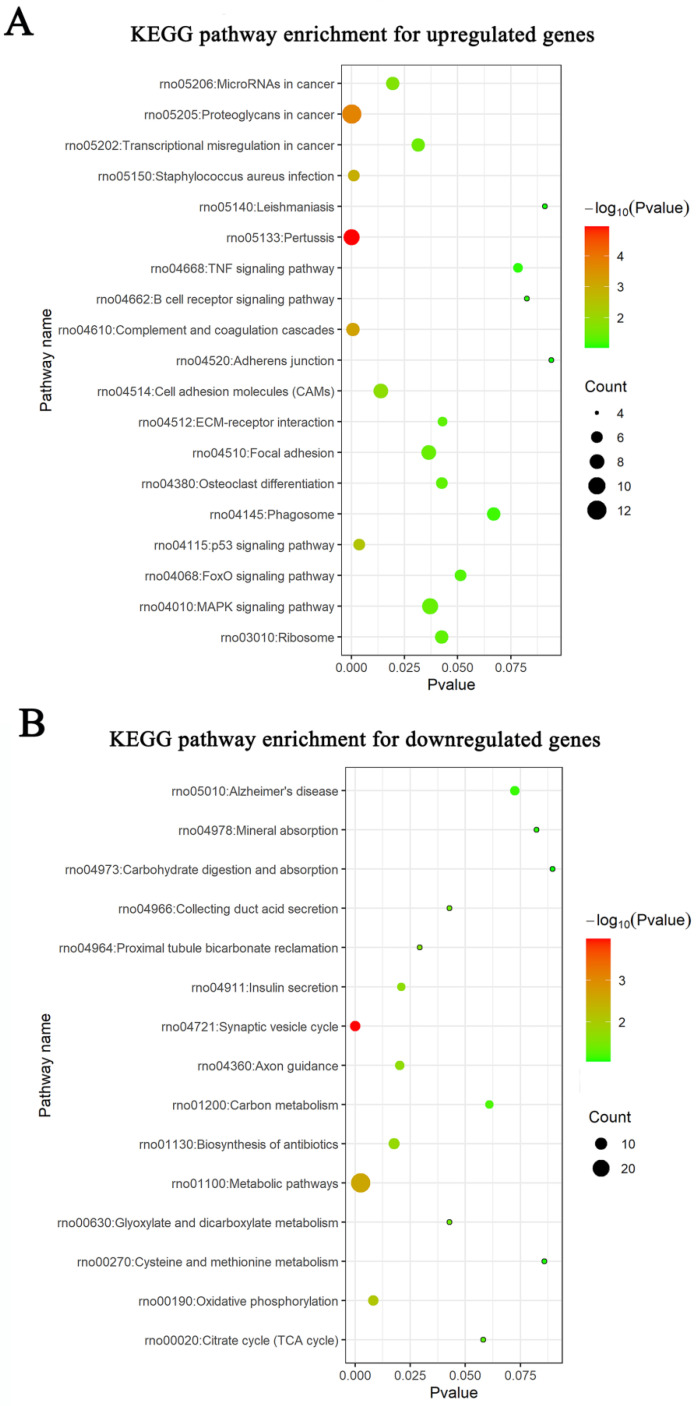
** KEGG pathways in DRG after PNI.** KEGG enrichment analyses were conducted for (A) up and (B) downregulated DEGs respectively.

Meta-analysis of five DRG gene expression databases revealed 211 upregulated mRNAs and 236 downregulated mRNAs (Figure 2) (Supplementary Table 1). GO analysis of these DEGs showed that functions of the upregulated genes in DRG after PNI were mainly concentrated in protein activity and cell secretion (GO:0032403~protein complex binding, GO:0050839~cell adhesion molecule binding, GO:0005515~protein binding, GO:0070062~ extracellular exosome). The downregulated gene functions were mainly concentrated in the activity of ion channels as well as synaptic and neuronal development (GO:0005216~ion channel activity, GO:0044456~synapse part, GO:0097458~neuron part, GO:0043005~neuron projection, GO:0007399~nervous system development) (Figure 3) (Supplementary Table 2). The KEGG enrichment analysis showed that the upregulated gene functions were concentrated in the TNF signaling pathway, cell adhesion molecules, p53 signaling pathway, etc., while the functions of downregulated genes were concentrated in the synaptic vesicle cycle, axon guidance, and citrate cycle, etc. (Figure 4).

**Figure 5:**
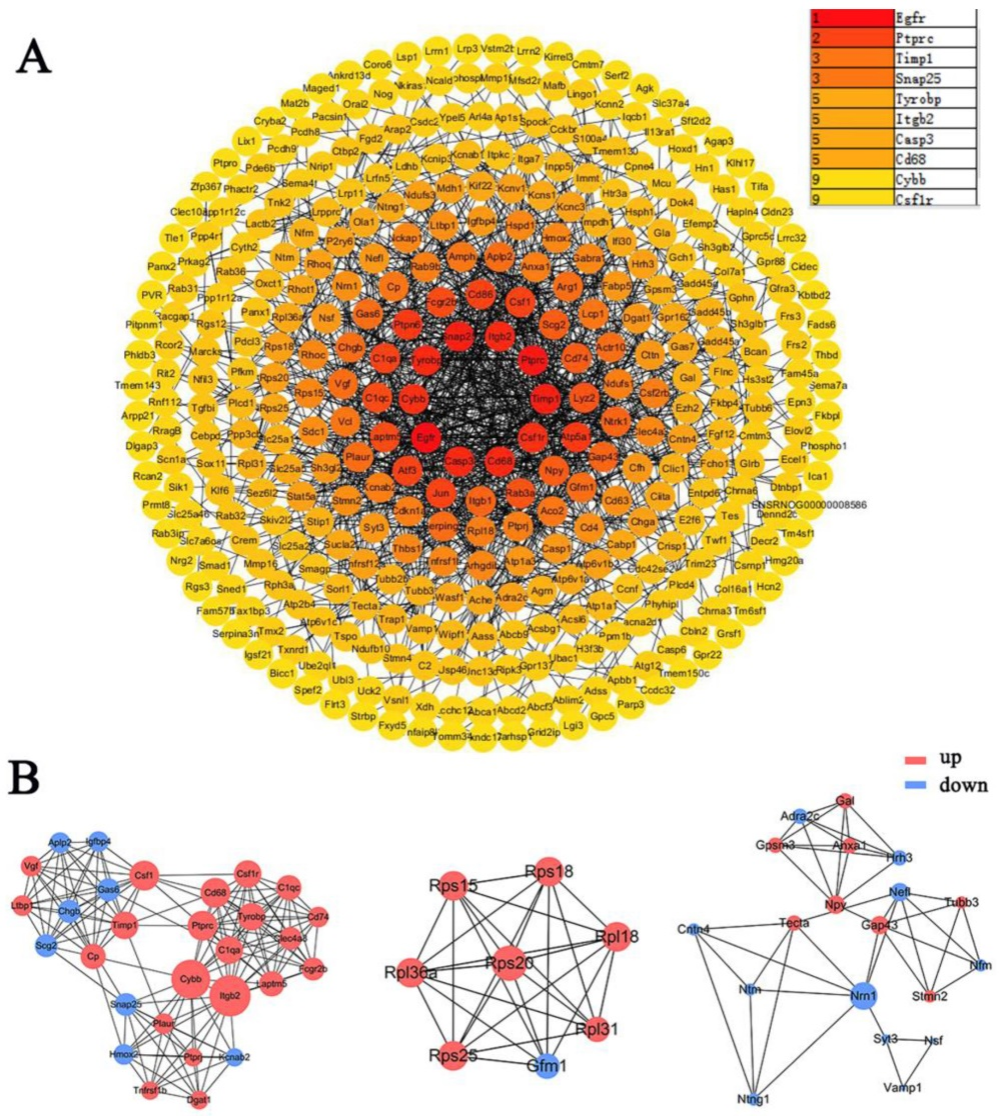
** PPI network and protein modules in DRG after PNI.** (A) All DEGs obtained from the meta-analysis were used to draw the PPI, and the interacting proteins were connected by lines. The darker the color of the node, the greater the interaction degree between the nodes. The top10 nodes with the highest degree were displayed in the upper right corner. (B) The top3 clustered functional modules in PPI, the red nodes represent upregulated proteins, and the blue nodes represent downregulated proteins. The size of one node is determined by the degree of its connection to other nodes.

The DEGs obtained from the meta-analysis were used to construct the protein-protein interaction (PPI) network, the depth of color was proportional to the degree of protein interaction (Figure 5A). The core proteins of the top3 functional modules clustered in PPI were Cybb, Rps20, and Nrn1 (Figure 5B). The top10 proteins with the highest interaction degree were Egfr, Ptprc, Timp1, Snap25, Tyrobp, Itgb2, Casp3, Cd68, Cybb, and Csf1r. Most of these proteins function in apoptosis, immunity, inflammation, and neurotransmitters (Table 3) (Supplementary Table 3).

### Potential microRNAs in DRG after peripheral nerve injury

**Figure 6:**
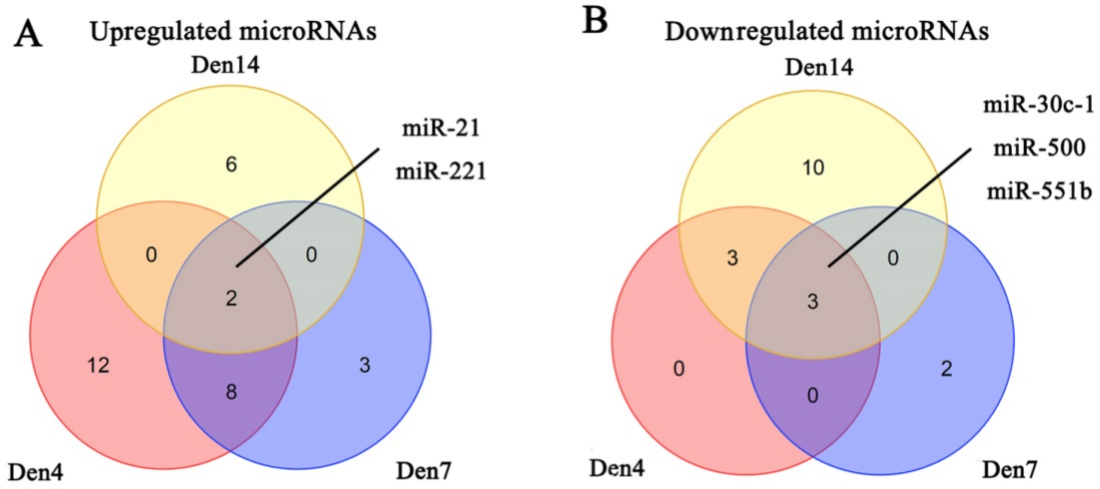
** Identification of microRNAs in DRG after PNI.** (A) 2 upregulated microRNAs and (B) 3 downregulated microRNAs were identified based on 3 DRG datasets after PNI. Den4, denervation for 4 days; Den7, denervation for 7 days; Den14, denervation for 14 days.

We obtained three microRNA expression databases of DRG after PNI 44. After taking the intersection of these databases, we found two upregulated microRNAs (miR-21, miR-221) and three downregulated microRNAs (miR-30C-1, miR-500, miR-551b) (Figure 6). We then predicted the target genes of these microRNAs and intersected these genes with the DEGs identified in our meta-analysis. Finally, we revealed 9 pairs of up-microRNA/down-gene interactions and 8 pairs of down-microRNA/up-gene interactions (Table 4).

### Validation of the hub mRNAs and miRNAs in vivo

**Figure 7:**
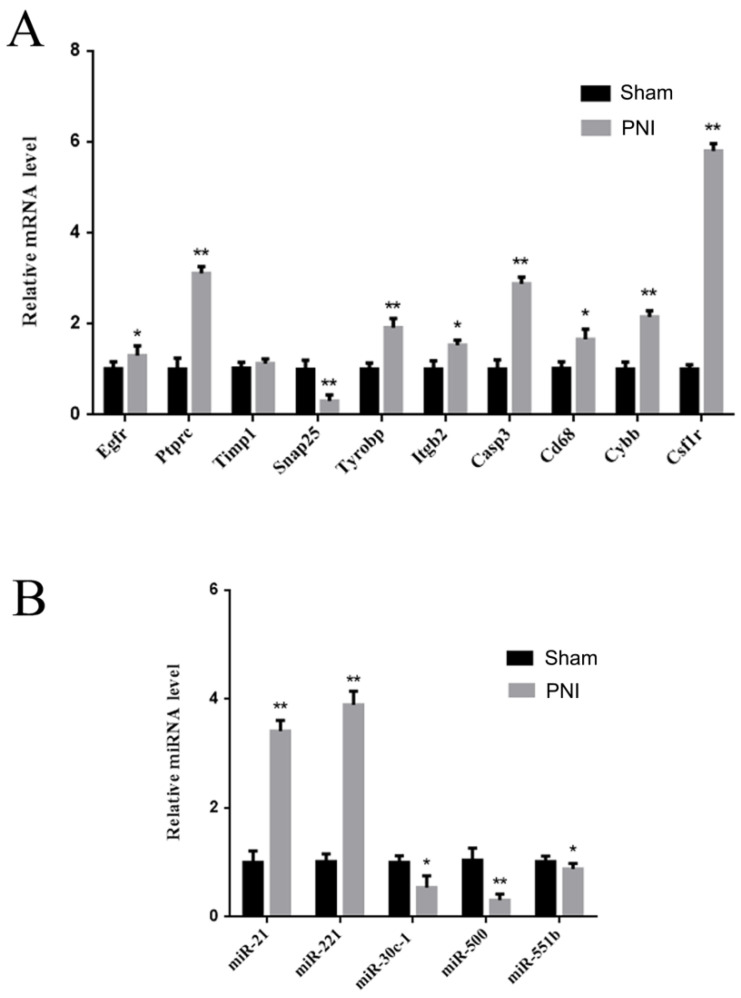
** Validation of the hub mRNAs and miRNAs expression level changes between the sham group and PNI group.** (A) The hub mRNA identified in PPI network. (B) The hub miRNA identified in meta analysis. Data are presented as the mean ± SD. * P< 0.05, ** P< 0.01 (Student's t-test). PNI: peripheral nerve injury.

To verify the identified hub mRNAs and miRNAs in vivo, DRGs from the sham group and PNI group were extracted. It was found by qRT-PCR that the expression levels of Egfr, Ptprc, Tyrobp, Itgb2, Casp3, Cd68, Cybb, and Csf1r increased, and the expression level of Snap25 decreased in the PNI group, which was consistent with the bioinformatics analysis. There was no difference in Timp1 expression between the sham group and the PNI group (Figure 7A). In addition, in the 1 week post-PNI group, the expression levels of miR-21 and miR-221 increased, and the expression levels of miR-30c-1, miR-500, and miR-551b decreased, which was in line with the results of bioinformatics analysis (Figure 7B).

### Construction of mTF-miRNA-gene-gTF regulatory network

**Figure 8:**
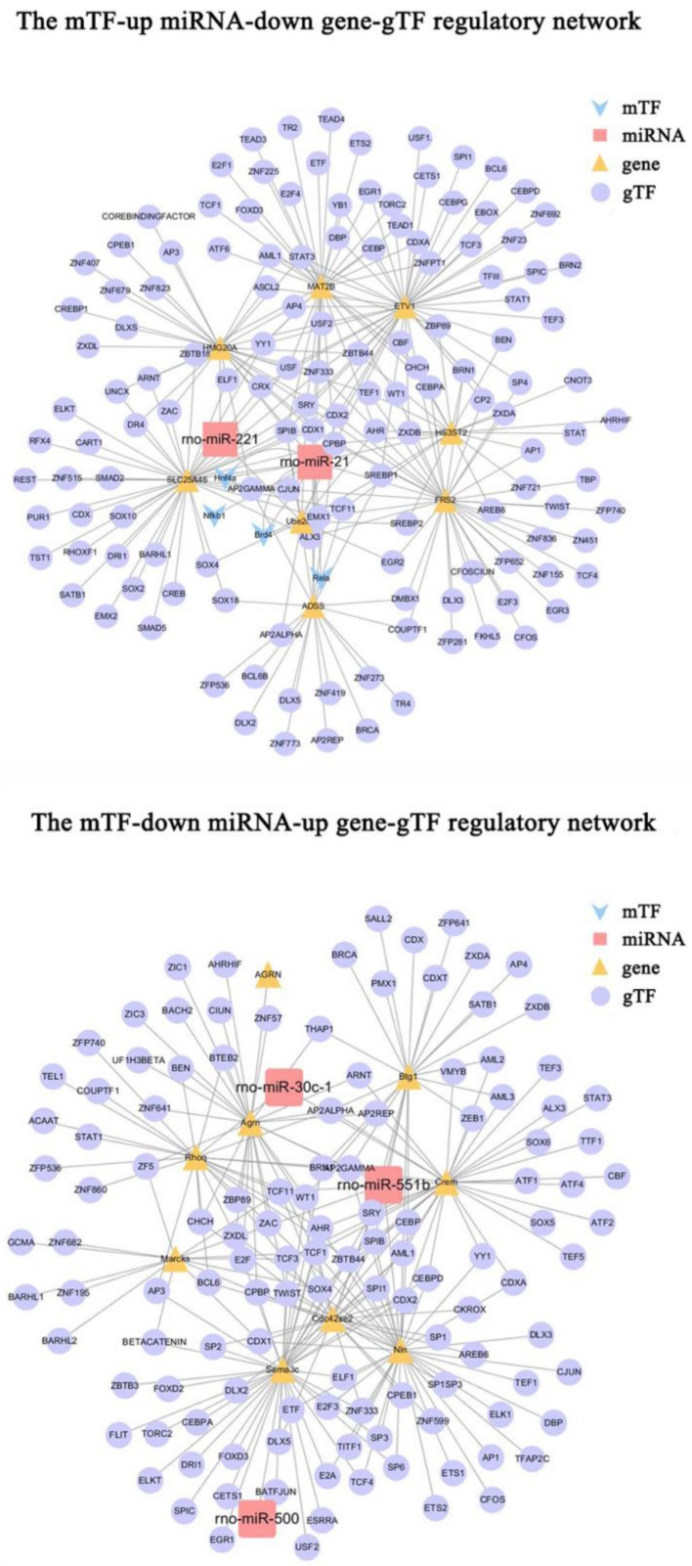
** The mTF-miRNA-gene-gTF regulatory network in DRG after PNI.** The mTF-miRNA-gene-gTF regulatory network of microRNA/gene interactions identified in DRG datasets after PNI obtained from Cytoscape software. mTF, TF associated with miRNAs; gTF, TF associated with genes.

To further investigate the regulatory mechanisms of microRNA-gene interactions in DRG after PNI, we constructed a regulatory network, including the above 9 pairs of up-microRNA/down-gene interactions and 8 pairs of down-microRNA/up-gene interactions, as well as the TF associated with these genes(gTF) and TF associated with these miRNAs(mTF) (Figure 8). Among them, miR-21 was regulated by most mTFs (Brd4, Hnf4a, Nfkb1, Rela), Etv1 was the downregulated gene with most gTFs, Sema3c was the upregulated gene with most gTFs, and Cpbp, Spib, Sry, Cdx1, and Ahr was the top5 gTF with most regulated genes (Table 5) (Supplementary Table 4-5).

### DRG transcriptome comparison between PNI and CCI

**Figure 9:**
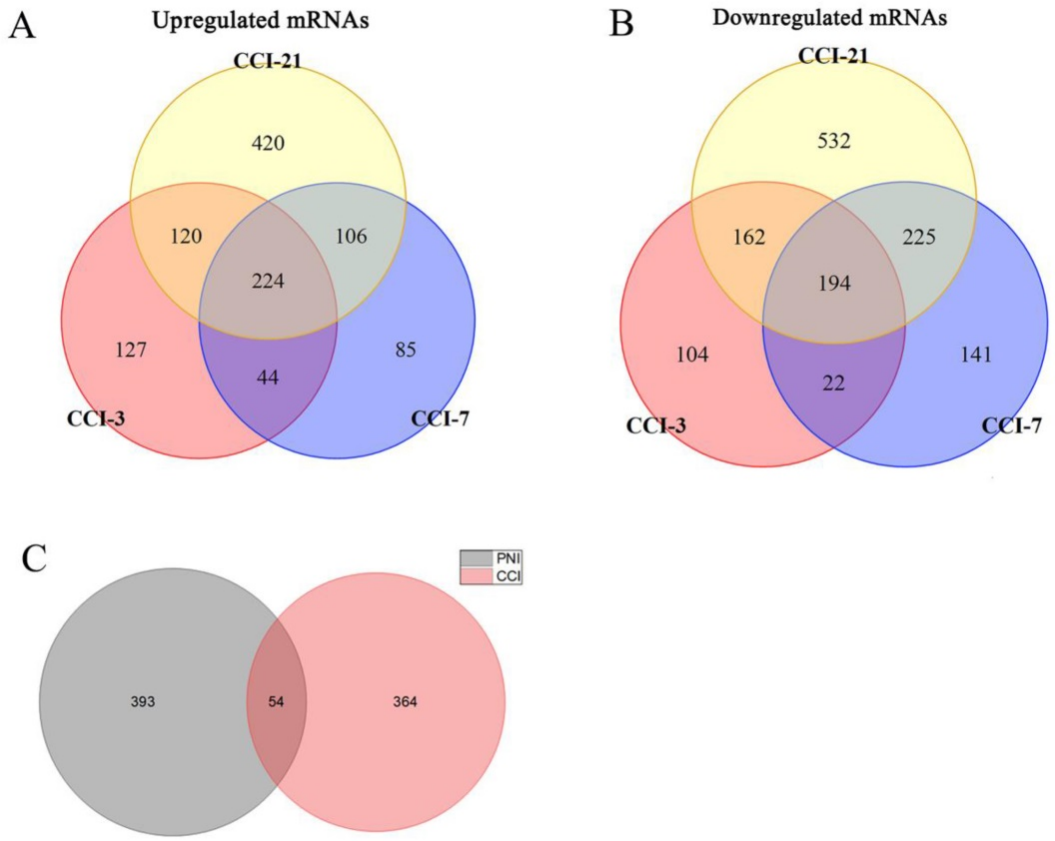
** Identification of DEGs in DRG after CCI and comparison between PNI and CCI.** (A) 224 upregulated mRNAs and (B) 194 downregulated mRNAs were identified based on 3 DRG datasets after CCI. (C) Venn diagram of PNI-DEGs and CCI-DEGs, PNI-DEGs are obtained from the meta-analysis in this study. CCI-3, chronic constriction injury for 3 days; CCI-7, chronic constriction injury for 7 days; CCI-21, chronic constriction injury for 21 days; PNI, peripheral nerve injury; CCI, chronic constriction injury.

**Figure 10:**
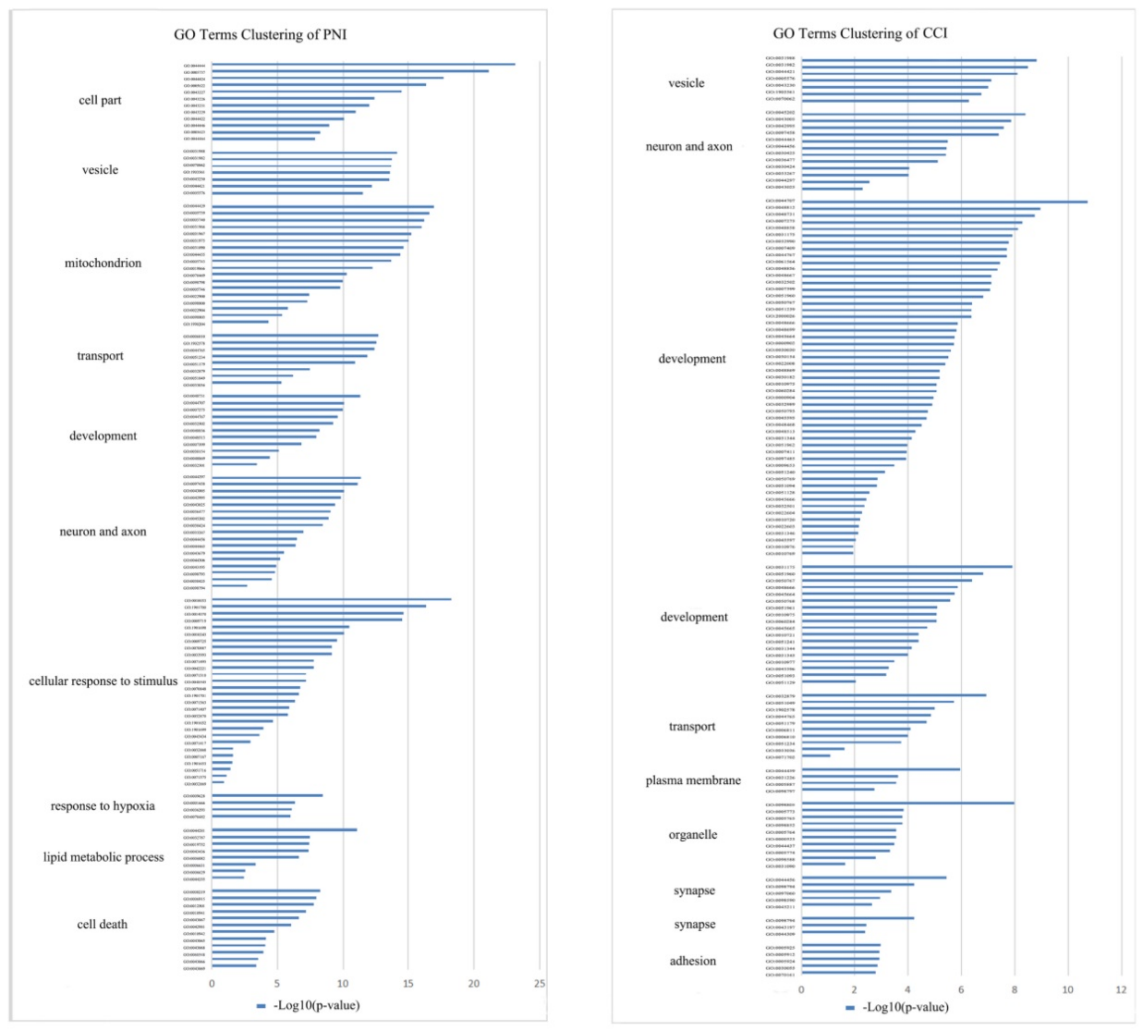
** GO terms clustering in DRG after PNI or CCI.** GO term enrichment analysis and clustering were performed for DEGs only in PNI and DEGs only in CCI, and the cluster groups with top 10 enrichment score were displayed. (A) GO terms clustering in DRG after PNI. (B) GO terms clustering in DRG after CCI. PNI, peripheral nerve injury; CCI, chronic constriction injury.

In order to compare the effects of PNI and CCI on DRG, we retrieved three gene expression datasets of DRG after CCI 45, and conducted meta-analysis of these datasets, obtaining 224 up-regulated genes and 194 down-regulated genes (Figure 9A, B) (Supplementary Table 6). Further, we compared PNI-DEGs and CCI-DEGs and found that only 54 genes intersected (Figure 9C). To explore the transcriptome features of the two model, GO term enrichment analysis and clustering were performed for DEGs only in PNI and DEGs only in CCI respectively (Figure 10) (Table 6-7) (Supplementary Table 7-8). The results showed that compared with CCI, PNI transcriptome changes were more focused on mitochondria, energy metabolism and apoptosis, while CCI transcriptome changes were more focused on tissue growth and development. To better understand the correlation of the two models with neuropathic pain, we compared the expression of pain-related genes in the DRG under the two models (Table 8). These pain-related genes include P2Xs, Transient Receptor Potential Receptors, NaVs and CaVs. The results showed that PNI caused more extensive changes in pain-related genes than CCI, and the related mechanisms of pain caused by the two may be different.

## Discussion

The change of DRG after nerve injury should be a process of interaction between DRG and injury stimuli. On the one hand, DRG transmits this injury signal to the central nervous system in the form of pain, and on the other hand, DRG will make some compliance with injury stimuli, including neuron damage, apoptosis and activation of regeneration program. The synthesis of various related factors increased and transported anterogradely to axons to promote axon regeneration 46-51. These processes involve complex gene regulation, which genes and microRNAs may play a major role in pain transmission and nerve regeneration is the focus of this study. Many studies have analyzed DRG transcriptome alterations after PNI, however, the integration of those databases is still lacking. As far as we know, this is the first meta-analysis to integrate multiple mRNAs and microRNAs expression databases of DRG transcriptome after PNI to identify common regulatory networks and molecular pathways.

First, we included five DRG gene expression databases after PNI and revealed 211 upregulated genes and 236 downregulated genes. We constructed the PPI network using the DEGs obtained from the meta-analysis and found that the top10 proteins with the highest interaction degree were Egfr, Ptprc, Timp1, Snap25, Tyrobp, Itgb2, Casp3, Cd68, Cybb, and Csf1r. It has been confirmed that Egfr, Timp1, Snap25, Casp-3, and Csf1r are involved in the regulation of neuropathic pain. Egfr is the member of the ERBB tyrosine kinase receptors family 52 which affects important receptors associated with pain regulation, including β adrenergic receptors 53, cannabinoid type 1 (CB1), opioid receptors 54, and transient receptor potential cation channel, subfamily V, member 1 (TRPV1) receptors 55. Timp1 can modulate 14 matrix metalloproteinases (MMPs) 56-58, which has been shown to prevent mechanical and thermal hypersensitivity after PNI 59, 60. Timp1 also can alleviate the development of inflammatory pain through cellular signaling mechanisms 61. Snap25, Syntaxin4, Vamp2, and MUNC18-1 are located at postsynaptic sites, and their complexes in the spinal dorsal horn can cause inflammatory pain 62. Casp-3 belongs to the caspase family, whose activation is the bifurcation point between cell plasticity and cell death 63. It was found that enhancing Casp-3 could alleviate peripheral pain hypersensitivity 64. Csf1r is a Csf receptor and regulates the development of the monocyte/macrophage lineage, which is involved in the control of neuropathic pain through the central action on microglia 65. Other genes are mostly involved in inflammation and immune regulation: Ptprc is an inflammation-related gene 66. As a signal transduction protein of several cell surface receptors, Tyrobp is a key regulator of the immune system 67. Itgb2 encodes CD18 protein, which is involved in immune regulation 68. Cd68, an inflammatory marker, is considered to be a selective marker for human monocytes and macrophages 69. Cybb is an immune regulator and encodes gp91-Phox protein that phagocytoses NADPH oxidase 70. As for nerve regeneration, Egfr plays a role in regulating neuronal and glial differentiation. In addition, it is involved in regeneration after injury and the development of neurodegenerative diseases 71. Timp1 was found to regulate the differentiation state and function of Schwann cells in neural regeneration 72. SNAP25b can promote axonal growth and spinal cord regeneration 73. Casp3 regulates nerve regeneration after stroke 74. The expression levels of these genes are significantly altered in DRG after PNI and may play an important role in the phenotypic alteration and functional regulation of DRG.

We further obtained three microRNA expression databases on DRG after PNI 44. After taking the intersection of the databases, we found two upregulated microRNAs (miR-21, miR-221) and three downregulated microRNAs (miR-30c-1, miR-500, miR-551b). MiR-21 is a key switch of inflammatory response and a key factor in tumor regulation 75, 76. However, recent studies found that miR-21 regulates neuronal excitability and DRG pain transmission via the regulation of Toll-like receptors 8 (TLR8) 77, 78. Meanwhile, miR-21 promotes Schwann cell proliferation and axon regeneration during the repair of injured nerves 79. The predictive targets of miR-21 also include Hmg20a, Slc25a46, Etv1, Adss, Mat2b, Frs2, and Hs3st2. Among which, Hmg20a is involved in neuronal differentiation and maturation 80. Frs2 is a lipid anchoring docking protein, which plays a role in signal transduction between neurons 81, 82. These genes may be important targets for miR-21 to regulate DRG after PNI. In addition, miR-221 can regulate neuropathic pain and promote Schwann cell proliferation 83, 84 and miR-500 can contribute to neuropathic pain by mediating the downregulation of GAD67 85. Studies found that the expression of miR-30c-5p was significantly altered in the thalamus and anterior cingulate gyrus of neuropathic pain animals, and its expression was up-regulated in the spinal cord, DRG, cerebrospinal fluid (CSF) and plasma of rats with sciatic nerve injury, and the expression of miR-30c-5p was associated with the severity of allodynia. Furthermore, its regulation has a relevant key role in the modulation of neuropathic pain 86, 87. MiR-551b-5p was found to directly target brain-derived neurotrophic factor (BDNF), thereby regulating neural structural and functional recovery 88.

To further investigate the regulatory mechanisms of microRNA-gene interactions, we constructed a regulatory network. Among them, miR-21 had the most mTFs (Brd4, Hnf4a, Nfkb1, Rela), Etv1 was the downregulated gene with the most gTFs, Sema3c was the upregulated gene with the most gTFs, and Cpbp was the gTF with the most regulated genes. Cpbp is a member of the Kruppel like factor (KLFs) protein family, which is involved in several key biological processes, such as proliferation, differentiation, metabolism, apoptosis, and inflammation. Cpbp can directly trans-activate TGFβ expression during tissue injury 89. At the same time, Cpbp regulates the expression of pro-inflammatory genes and inflammatory macrophage polarization by cooperating with NF-κβ 90, 91. This may be the primary mechanism by which Cpbp regulates related genes as a gTF.

CCI is a classic model of neuropathic pain that produces spontaneous pain characteristics with relatively minor nerve damage by placing a loose constrictive ligation around the common sciatic nerve 29. we compared the DRG transcriptome after PNI to that of CCI and found that, PNI transcriptome changes were more focused on mitochondria, energy metabolism and apoptosis, while CCI transcriptome changes were more focused on tissue growth and development. By comparing the expression of pain-related genes in the DRG under the two models, we found PNI caused more extensive changes in pain-related genes than CCI. This suggests that nerve injury will cause more damage to DRG than simple pain, which is mainly manifested in the change of mitochondrial function and the initiation of apoptosis mechanism. Furthermore, the related mechanisms of pain caused by the two may be different.

## Conclusions

In conclusion, this study is the first to comprehensively integrate and analyze the DRG-related mRNA expression and microRNA expression databases after PNI. First, we identified several genes playing an important role in the regulation of neuropathic pain and nerve regeneration, including Egfr, Ptprc, Timp1, Snap25, Tyrobp, Itgb2, Casp3, Cd68, Cybb, and Csf1r. Second, we further predicted new microRNA-mRNA interactions, such as miR-21/Hmg20a, miR-221/Ube2ql1, miR-30c-1/Rhoq, miR-500/Sema3c, and miR-551b/Cdc42se2, which may be important regulatory nodes. And we constructed the mTF-miRNA-gene-gTF regulatory network to further explore the molecular mechanism in DRG. Third, we compared the DRG transcriptome after PNI to that of CCI and found that PNI would cause greater damage stimulation to DRG than CCI, and the related mechanisms of pain caused by the two may be different.

## Supplementary Material

Supplementary tables.

## Figures and Tables

**Table 1:** primer sequences.

mRNA/miRNA	Sequence
Gapdh	forward primer: 5'-CTT CTC TTG TGA CAA AG TGG-3' reverse primer: 5'-GTA GAC TCC ACG ACA TAC TC-3'
Egfr	forward primer: 5'- GTC CGG GCA GCC CCC-3' reverse primer: 5'- TAG CTT TTG CTC TTT TAT TAA GTT ACT GTT-3'
Ptprc	forward primer: 5'-CGA ACA AAT CCT CAG CCT A-3' reverse primer: 5'- CCT CCC CTT TCC ATG TG-3'
Timp1	forward primer: 5'-ACA GCT TTC TGC AAC TCG GA -3' reverse primer: 5'-CGG AAA CCT GTG GCA TTT CC-3'
Snap25	forward primer: 5'- GAG TCC CTG GAA AGC ACC-3' reverse primer: 5'-GGC ATC GTT TGT TAC CCT-3'
Tyrobp	forward primer: 5'-CAG GCC CAG AGT GAC AAT TAC C-3' reverse primer: 5'-ATG AGC AGA GTC AGC ACC AAG-3'
Itgb2	forward primer: 5'-CAT CTG GCC CTT CTC TCC AC-3' reverse primer: 5'-ACT TGG TGC ATT CCT CGG AC-3'
Casp3	forward primer: 5'- GCC GAA ACT CTT CAT CAT TCA GG-3' reverse primer: 5'- CAT ATC ATC GTC AGT TCC ACT GTC-3'
Cd68	forward primer: 5'-CCT GAC CCA GGG TGG AAA AA-3' reverse primer: 5'-TGA GAG AGC CAA GTG GGG AT-3'
Cybb	forward primer: 5'-GTT TGC CGG AAA CCC TCC TA-3' reverse primer: 5'-CCT TCT GCT GAG ATC GCC AA-3'
Csf1r	forward primer: 5'- GCG AGG GTT CAT TAT CCA CAA G-3' reverse primer: 5'-TCA CCA GCT TAG TAG GTT CCA ATA T-3'

**Table 2:** Description of public data sets included in the meta-analysis.

Number	Gene Expression Platforms	Surgery	Species	Intervention duration	Ref.
1	Illumina HiSeq 2000	pSNL	Rattus norvegicus	7 days	GSE117526^42^
2	Agilent-014879 Whole Rat Genome Microarray	Sciatic nerve resection	Rattus norvegicus	4 days	GSE30165^41^
3	Agilent-014879 Whole Rat Genome Microarray	Sciatic nerve resection	Rattus norvegicus	7 days	GSE30165^41^
4	Agilent-014879 Whole Rat Genome Microarray	Sciatic nerve resection	Rattus norvegicus	14 days	GSE30165^41^
5	Affymetrix GeneChip Mouse Transcriptome Array 1.0	pSNL	Mus Musculus	16 days	E-MTAB-6864^43^

pSNL, partial sciatic nerve ligation; Ref, Reference.

**Table 3:** Description of top10 proteins in PPI.

Official Symbol	Molecular function	Biological process	Location
Egfr	Developmental protein, Host cell receptor for virus entry, Kinase	Host-virus interaction	Intracellular, Membrane, Secreted
Ptprc	Protein tyrosine phosphatase, receptor type C	cell growth, differentiation, mitosis	Intracellular, Membrane
Timp1	Growth factor, Metalloenzyme inhibitor	cell differentiation, migration and cell death	Intracellular, Secreted
Snap25	Synaptosome associated protein	Neurotransmitter release	Intracellular
Tyrobp	TYRO protein tyrosine kinase binding protein	Immunity	Membrane
Itgb2	Integrin, Receptor	Cell adhesion, Phagocytosis	Intracellular, Membrane
Casp3	Hydrolase, Protease, Thiol protease	Apoptosis	Intracellular
Cd68	Binds to tissue- and organ-specific lectins or selectins	Phagocytic activities of tissue macrophages	Intracellular, Membrane
Cybb	Ion channel, Oxidoreductase	Electron transport, Ion transport, Transport	Membrane
Csf1r	Kinase, Receptor, Transferase, Tyrosine-protein kinase	Immunity, Inflammatory response, Innate immunity	Intracellular, Membrane

PPI, protein-protein interaction.

**Table 4:** MicroRNA/gene interactions.

Up-MicroRNA / Down-gene	Down-MicroRNA / Up-gene
miR-21 / Hmg20a	miR-30c-1 / Rhoq
miR-21 / Slc25a46	miR-30c-1 / Crem
miR-21 / Etv1	miR-500 / Sema3c
miR-21 / Adss	miR-551b / Cdc42se2
miR-21 / Mat2b	miR-551b / Agrn
miR-21 / Frs2	miR-551b / Nln
miR-21 / Hs3st2	miR-551b / Btg1
miR-221 / Ube2ql1	miR-551b / Marcks
miR-221 / Hmg20a	

**Table 5:** Top5 gTF regulating most DEGs.

gTF	DEGs	Gene counts	miRNA targeting DEGs
Cpbp	Hmg20a, Slc25a46, Etv1, Adss, Mat2b, Frs2, Hs3St2, Ube2ql1, Sema3c, Cdc42se2, Agrn, Nln, Marcks	13	miR-500, miR-551b, miR-21, miR-221
Spib	Hmg20a, Slc25a46, Etv1, Adss Mat2b, Ube2ql1, Crem, Sema3c, Cdc42Se2, Nln, Btg1	11	miR-30c-1, miR-500, miR-21, miR-551b, miR-221
Sry	Hmg20a, Slc25a46, Etv1, Mat2b, Frs2, Ube2ql1, Crem, Sema3c, Cdc42se2, Btg1	10	miR-30c-1, miR-500, miR-21, miR-551b, miR-221
Cdx1	Hmg20a, Slc25a46, Etv1, Mat2b, Frs2, Cdc42se2, Nln, Marcks, Adss	9	miR-21, miR-551b, miR-221
Ahr	Mat2b, Hs3st2, Ube2ql1, Rhoq, Crem, Sema3c, Agrn, Nln	8	miR-30c-1, miR-500, miR-21, miR-551b, miR-221
			

gTF, TF associated with these genes; DEG, Differently Expressed Gene.

**Table 6:** GO terms clustering in DRG after PNI.

Annotation Cluster	Enrichment Score	Term
Cell part	13.62	GO:0044444, GO:0005737, GO:0044424, GO:0005622, GO:0043227, GO:0043226, GO:0043231, GO:0043229, GO:0044422…
Vesicle	13.20	GO:0031988, GO:0031982, GO:0070062, GO:1903561, GO:0043230, GO:0044421, GO:0005576
Mitochondrion	11.74	GO:0044429, GO:0005739, GO:0005740, GO:0031966, GO:0031967, GO:0031975, GO:0031090, GO:0044455, GO:0005743…
Transport	9.91	GO:0006810, GO:1902578, GO:0044765, GO:0051234, GO:0051179, GO:0032879, GO:0051049, GO:0033036
Development	7.85	GO:0048731, GO:0044707, GO:0007275, GO:0044767, GO:0032502, GO:0048856, GO:0048513, GO:0007399, GO:0030154…
Neuron and axon	7.40	GO:0044297, GO:0097458, GO:0043005, GO:0042995, GO:0043025, GO:0036477, GO:0045202, GO:0030424, GO:0033267…
Cellular response to stimulus	7.03	GO:0010033, GO:1901700, GO:0014070, GO:0009719, GO:1901698, GO:0010243, GO:0009725, GO:0070887, GO:0033993…
Response to hypoxia	6.72	GO:0009628, GO:0001666, GO:0036293, GO:0070482
Lipid metabolic process	6.05	GO:0044281, GO:0032787, GO:0019752, GO:0043436, GO:0006082, GO:0006631, GO:0006629, GO:0044255
Cell death	5.64	GO:0008219, GO:0006915, GO:0012501, GO:0010941, GO:0043067, GO:0042981, GO:0010942, GO:0043065, GO:0043068…

**Table 7:** GO terms clustering in DRG after CCI.

Annotation Cluster	Enrichment Score	Term
Vesicle	7.51	GO:0031988, GO:0031982, GO:0044421, GO:0005576, GO:0043230, GO:1903561, GO:0070062
Neuron and axon	5,47	GO:0045202, GO:0043005, GO:0042995, GO:0097458, GO:0044463, GO:0044456, GO:0030425, GO:0036477, GO:0030424…
Development	5.22	GO:0044707, GO:0048812, GO:0048731, GO:0007275, GO:0048858, GO:0031175, GO:0032990, GO:0007409, GO:0044767…
Development	4.84	GO:0031175, GO:0051960, GO:0050767, GO:0048666, GO:0045664, GO:0050768, GO:0051961, GO:0010975, GO:0060284…
Transport	4.17	GO:0032879, GO:0051049, GO:1902578, GO:0044765, GO:0051179, GO:0006811, GO:0006810, GO:0051234, GO:0033036…
Plasma membrane	3.96	GO:0044459, GO:0031226, GO:0005887, GO:0098797
Organelle	3.77	GO:0098805, GO:0005773, GO:0005765, GO:0098852, GO:0005764, GO:0000323, GO:0044437, GO:0005774, GO:0098588…
Synapse	3.72	GO:0044456, GO:0098794, GO:0097060, GO:0098590, GO:0045211
Synapse	3.01	GO:0098794, GO:0043197, GO:0044309
Adhesion	2.89	GO:0005925, GO:0005912, GO:0005924, GO:0030055, GO:0070161

CCI, chronic constriction injury.

**Table 8:** Molecular mediators of neuropathic pain in PNI and CCI models.

Injury Model	Intervention duration	P2Xs	TRPs	NaVs	CaVs
PNI	1-7 days	P2rx2, P2rx3, P2rx4, P2rx5, P2rx6	Trpa1, Trpm1, Trpm2, Trpm4, Trpm5, Trpm6, Trpm8, Trpv1, Trpv5	Scn1a, Scn2a1, Scn3a, Scn5a, Scn7a, Scn8a, Scn9a, Scn10a, Scn11a	Cacna1i
	7-21days	P2rx1, P2rx2, P2rx3, P2rx4, P2rx5, P2rx6, P2rx7	Trpa1, Trpm1, Trpm2, Trpm3, Trpm4, Trpm5, Trpm6, Trpm7, Trpm8, Trpv1, Trpv2, Trpv3, Trpv4, Trpv5, Trpv6	Scn1a, Scn2a1, Scn3a, Scn4a, Scn5a, Scn7a, Scn8a, Scn9a	Cacna1h, Cacna1i
CCI	1-7 days	P2rx2, P2rx5, P2rx6		Scn1a, Scn4a, Scn7a, Scn9a, Scn11a	
	7-21days	P2rx2		Scn1a, Scn2a, Scn3a, Scn9, Scn11a	

## Abbreviations

DRG: dorsal root ganglia
DEGs: differentially expressed genes
gTFs: transcription factors associated with genes
mTFs: TFs associated with these microRNAs
CCI: chronic constriction injury
P2Xs: Purinergic P2X Receptors
NaVs: Voltage-Gated Sodium Channels
CaVs: Voltage-Gated Calcium Channels
GAP: growth associated protein
SCG10: superior cervical ganglion 10
SPRR1A: small proline-rich repeat protein 1A
GO: Gene Ontology
KEGG: Kyoto Encyclopedia of Genes and Genomes
PPI: protein-protein interaction
CB1: cannabinoid type 1
TRPV1: transient receptor potential cation channel, subfamily V, member 1
MMPs: matrix metalloproteinases
TLR8: Toll-like receptors 8
KLFs: Kruppel like factor

## References

[1] Hogan QH (2010). Labat lecture: the primary sensory neuron: where it is, what it does, and why it matters. Reg Anesth Pain Med.

[2] Pokhilko A, Nash A, Cader MZ (2020). Common transcriptional signatures of neuropathic pain. Pain.

[3] Martin SL, Reid AJ, Verkhratsky A, Magnaghi V, Faroni A (2019). Gene expression changes in dorsal root ganglia following peripheral nerve injury: roles in inflammation, cell death and nociception. Neural Regen Res.

[4] Smith DS, Skene JH (1997). A transcription-dependent switch controls competence of adult neurons for distinct modes of axon growth. J Neurosci.

[5] Weng YL, Wang X, An R, Cassin J, Vissers C, Liu Y (2018). Epitranscriptomic m(6)A Regulation of Axon Regeneration in the Adult Mammalian Nervous System. Neuron.

[6] Mar FM, Bonni A, Sousa MM (2014). Cell intrinsic control of axon regeneration. EMBO Rep.

[7] Mar FM, Simoes AR, Rodrigo IS, Sousa MM (2016). Inhibitory Injury Signaling Represses Axon Regeneration After Dorsal Root Injury. Mol Neurobiol.

[8] Yeh TY, Luo IW, Hsieh YL, Tseng TJ, Chiang H, Hsieh ST (2020). Peripheral Neuropathic Pain: From Experimental Models to Potential Therapeutic Targets in Dorsal Root Ganglion Neurons. Cells.

[9] Chen L, Liu YW, Yue K, Ru Q, Xiong Q, Ma BM (2016). Differential expression of ATP-gated P2X receptors in DRG between chronic neuropathic pain and visceralgia rat models. Purinergic Signal.

[10] Leng C, Chen L, Li C (2019). Alteration of P2X1-6 receptor expression in retrograde Fluorogold-labeled DRG neurons from rat chronic neuropathic pain model. Biomed Rep.

[11] Barclay J, Patel S, Dorn G, Wotherspoon G, Moffatt S, Eunson L (2002). Functional downregulation of P2X3 receptor subunit in rat sensory neurons reveals a significant role in chronic neuropathic and inflammatory pain. J Neurosci.

[12] Naziroglu M, Braidy N (2017). Thermo-Sensitive TRP Channels: Novel Targets for Treating Chemotherapy-Induced Peripheral Pain. Front Physiol.

[13] Wang C, Gu L, Ruan Y, Geng X, Xu M, Yang N (2019). Facilitation of MrgprD by TRP-A1 promotes neuropathic pain. FASEB J.

[14] Schwartz ES, Christianson JA, Chen X, La JH, Davis BM, Albers KM (2011). Synergistic role of TRPV1 and TRPA1 in pancreatic pain and inflammation. Gastroenterology.

[15] Story GM (2006). The emerging role of TRP channels in mechanisms of temperature and pain sensation. Curr Neuropharmacol.

[16] Su L, Shu R, Song C, Yu Y, Wang G, Li Y (2017). Downregulations of TRPM8 expression and membrane trafficking in dorsal root ganglion mediate the attenuation of cold hyperalgesia in CCI rats induced by GFRalpha3 knockdown. Brain Res Bull.

[17] Black JA, Dib-Hajj S, McNabola K, Jeste S, Rizzo MA, Kocsis JD (1996). Spinal sensory neurons express multiple sodium channel alpha-subunit mRNAs. Brain Res Mol Brain Res.

[18] Brouwer BA, Merkies IS, Gerrits MM, Waxman SG, Hoeijmakers JG, Faber CG (2014). Painful neuropathies: the emerging role of sodium channelopathies. J Peripher Nerv Syst.

[19] Kang XJ, Chi YN, Chen W, Liu FY, Cui S, Liao FF (2018). Increased expression of CaV3.2 T-type calcium channels in damaged DRG neurons contributes to neuropathic pain in rats with spared nerve injury. Mol Pain.

[20] Shin JB, Martinez-Salgado C, Heppenstall PA, Lewin GR (2003). A T-type calcium channel required for normal function of a mammalian mechanoreceptor. Nat Neurosci.

[21] Dubovy P, Klusakova I, Hradilova-Svizenska I, Joukal M (2018). Expression of Regeneration-Associated Proteins in Primary Sensory Neurons and Regenerating Axons After Nerve Injury-An Overview. Anat Rec (Hoboken).

[22] Murillo B, Mendes Sousa M (2018). Neuronal Intrinsic Regenerative Capacity: The Impact of Microtubule Organization and Axonal Transport. Dev Neurobiol.

[23] Bartel DP (2009). MicroRNAs: target recognition and regulatory functions. Cell.

[24] Chattopadhyay M, Zhou Z, Hao S, Mata M, Fink DJ (2012). Reduction of voltage gated sodium channel protein in DRG by vector mediated miRNA reduces pain in rats with painful diabetic neuropathy. Mol Pain.

[25] Im YB, Jee MK, Choi JI, Cho HT, Kwon OH, Kang SK (2012). Molecular targeting of NOX4 for neuropathic pain after traumatic injury of the spinal cord. Cell Death Dis.

[26] Willemen HL, Huo XJ, Mao-Ying QL, Zijlstra J, Heijnen CJ, Kavelaars A (2012). MicroRNA-124 as a novel treatment for persistent hyperalgesia. J Neuroinflammation.

[27] Wang T, Li B, Wang Z, Yuan X, Chen C, Zhang Y (2019). miR-155-5p Promotes Dorsal Root Ganglion Neuron Axonal Growth in an Inhibitory Microenvironment via the cAMP/PKA Pathway. Int J Biol Sci.

[28] Chen HP, Zhou W, Kang LM, Yan H, Zhang L, Xu BH (2014). Intrathecal miR-96 inhibits Nav1.3 expression and alleviates neuropathic pain in rat following chronic construction injury. Neurochem Res.

[29] Bennett GJ, Xie YK (1988). A peripheral mononeuropathy in rat that produces disorders of pain sensation like those seen in man. Pain.

[30] Moher D, Liberati A, Tetzlaff J, Altman DG, Group P (2010). Preferred reporting items for systematic reviews and meta-analyses: the PRISMA statement. Int J Surg.

[31] Ramasamy A, Mondry A, Holmes CC, Altman DG (2008). Key issues in conducting a meta-analysis of gene expression microarray datasets. PLoS Med.

[32] von Mering C, Huynen M, Jaeggi D, Schmidt S, Bork P, Snel B (2003). STRING: a database of predicted functional associations between proteins. Nucleic Acids Res.

[33] Kohl M, Wiese S, Warscheid B (2011). Cytoscape: software for visualization and analysis of biological networks. Methods Mol Biol.

[34] Chin CH, Chen SH, Wu HH, Ho CW, Ko MT, Lin CY (2014). cytoHubba: identifying hub objects and sub-networks from complex interactome. BMC Syst Biol.

[35] Agarwal V, Bell GW, Nam JW, Bartel DP (2015). Predicting effective microRNA target sites in mammalian mRNAs. Elife.

[36] Chen Y, Wang X (2020). miRDB: an online database for prediction of functional microRNA targets. Nucleic Acids Res.

[37] Maragkakis M, Vergoulis T, Alexiou P, Reczko M, Plomaritou K, Gousis M (2011). DIANA-microT Web server upgrade supports Fly and Worm miRNA target prediction and bibliographic miRNA to disease association. Nucleic Acids Res.

[38] Schmittgen TD, Livak KJ (2008). Analyzing real-time PCR data by the comparative C(T) method. Nat Protoc.

[39] Fogel GB, Weekes DG, Varga G, Dow ER, Craven AM, Harlow HB (2005). A statistical analysis of the TRANSFAC database. Biosystems.

[40] Tong Z, Cui Q, Wang J, Zhou Y (2019). TransmiR v2.0: an updated transcription factor-microRNA regulation database. Nucleic Acids Res.

[41] Li S, Liu Q, Wang Y, Gu Y, Liu D, Wang C (2013). Differential gene expression profiling and biological process analysis in proximal nerve segments after sciatic nerve transection. PLoS One.

[42] Sun W, Kou D, Yu Z, Yang S, Jiang C, Xiong D (2020). A Transcriptomic Analysis of Neuropathic Pain in Rat Dorsal Root Ganglia Following Peripheral Nerve Injury. Neuromolecular medicine.

[43] Bangash MA, Alles SRA, Santana-Varela S, Millet Q, Sikandar S, de Clauser L (2018). Distinct transcriptional responses of mouse sensory neurons in models of human chronic pain conditions. Wellcome Open Res.

[44] Yu B, Zhou S, Qian T, Wang Y, Ding F, Gu X (2011). Altered microRNA expression following sciatic nerve resection in dorsal root ganglia of rats. Acta biochimica et biophysica Sinica.

[45] Costigan M, Belfer I, Griffin RS, Dai F, Barrett LB, Coppola G (2010). Multiple chronic pain states are associated with a common amino acid-changing allele in KCNS1. Brain.

[46] Lawrence T (2009). The nuclear factor NF-kappaB pathway in inflammation. Cold Spring Harb Perspect Biol.

[47] Schafers M, Geis C, Brors D, Yaksh TL, Sommer C (2002). Anterograde transport of tumor necrosis factor-alpha in the intact and injured rat sciatic nerve. J Neurosci.

[48] Uceyler N, Tscharke A, Sommer C (2007). Early cytokine expression in mouse sciatic nerve after chronic constriction nerve injury depends on calpain. Brain Behav Immun.

[49] Abe S, Mizusawa I, Kanno K, Yabashi A, Suto M, Kuraya M (2003). Nitric oxide synthase expressions in rat dorsal root ganglion after a hind limb tourniquet. Neuroreport.

[50] Momeni HR, Soleimani Mehranjani M, Shariatzadeh MA, Haddadi M (2013). Caspase-mediated apoptosis in sensory neurons of cultured dorsal root Ganglia in adult mouse. Cell J.

[51] Vigneswara V, Berry M, Logan A, Ahmed Z (2013). Caspase-2 is upregulated after sciatic nerve transection and its inhibition protects dorsal root ganglion neurons from apoptosis after serum withdrawal. PLoS One.

[52] Citri A, Yarden Y (2006). EGF-ERBB signalling: towards the systems level. Nat Rev Mol Cell Biol.

[53] Liu X, Wu WK, Yu L, Li ZJ, Sung JJ, Zhang ST (2008). Epidermal growth factor-induced esophageal cancer cell proliferation requires transactivation of beta-adrenoceptors. J Pharmacol Exp Ther.

[54] Chen Y, Long H, Wu Z, Jiang X, Ma L (2008). EGF transregulates opioid receptors through EGFR-mediated GRK2 phosphorylation and activation. Mol Biol Cell.

[55] Yang H, Wang Z, Capo-Aponte JE, Zhang F, Pan Z, Reinach PS (2010). Epidermal growth factor receptor transactivation by the cannabinoid receptor (CB1) and transient receptor potential vanilloid 1 (TRPV1) induces differential responses in corneal epithelial cells. Exp Eye Res.

[56] Gardner J, Ghorpade A (2003). Tissue inhibitor of metalloproteinase (TIMP)-1: the TIMPed balance of matrix metalloproteinases in the central nervous system. J Neurosci Res.

[57] Baker AH, Edwards DR, Murphy G (2002). Metalloproteinase inhibitors: biological actions and therapeutic opportunities. J Cell Sci.

[58] Nagase H, Visse R, Murphy G (2006). Structure and function of matrix metalloproteinases and TIMPs. Cardiovasc Res.

[59] Kawasaki Y, Xu ZZ, Wang X, Park JY, Zhuang ZY, Tan PH (2008). Distinct roles of matrix metalloproteases in the early- and late-phase development of neuropathic pain. Nat Med.

[60] Martinho FC, Teixeira FF, Cardoso FG, Ferreira NS, Nascimento GG, Carvalho CA (2016). Clinical Investigation of Matrix Metalloproteinases, Tissue Inhibitors of Matrix Metalloproteinases, and Matrix Metalloproteinase/Tissue Inhibitors of Matrix Metalloproteinase Complexes and Their Networks in Apical Periodontitis. J Endod.

[61] Knight BE, Kozlowski N, Havelin J, King T, Crocker SJ, Young EE (2019). TIMP-1 Attenuates the Development of Inflammatory Pain Through MMP-Dependent and Receptor-Mediated Cell Signaling Mechanisms. Front Mol Neurosci.

[62] Duan XL, Guo Z, He YT, Li YX, Liu YN, Bai HH (2020). SNAP25/syntaxin4/VAMP2/Munc18-1 Complexes in Spinal Dorsal Horn Contributed to Inflammatory Pain. Neuroscience.

[63] Snigdha S, Smith ED, Prieto GA, Cotman CW (2012). Caspase-3 activation as a bifurcation point between plasticity and cell death. Neurosci Bull.

[64] Wang YJ, Liu MG, Wang JH, Cao W, Wu C, Wang ZY (2020). Restoration of Cingulate Long-Term Depression by Enhancing Non-apoptotic Caspase 3 Alleviates Peripheral Pain Hypersensitivity. Cell Rep.

[65] Saleh R, Lee MC, Khiew SH, Louis C, Fleetwood AJ, Achuthan A (2018). CSF-1 in Inflammatory and Arthritic Pain Development. J Immunol.

[66] D'Angelo A, Sobhani N, Roviello G, Bagby S, Bonazza D, Bottin C (2019). Tumour infiltrating lymphocytes and immune-related genes as predictors of outcome in pancreatic adenocarcinoma. PLoS One.

[67] Ma J, Jiang T, Tan L, Yu JT (2015). TYROBP in Alzheimer's disease. Mol Neurobiol.

[68] Gomez JC, Doerschuk CM (2010). The role of CD18 in the production and release of neutrophils from the bone marrow. Lab Invest.

[69] Gottfried E, Kunz-Schughart LA, Weber A, Rehli M, Peuker A, Muller A (2008). Expression of CD68 in non-myeloid cell types. Scand J Immunol.

[70] Tarazona-Santos E, Bernig T, Burdett L, Magalhaes WC, Fabbri C, Liao J (2008). CYBB, an NADPH-oxidase gene: restricted diversity in humans and evidence for differential long-term purifying selection on transmembrane and cytosolic domains. Hum Mutat.

[71] Romano R, Bucci C (2020). Role of EGFR in the Nervous System. Cells.

[72] Kim Y, Remacle AG, Chernov AV, Liu H, Shubayev I, Lai C (2012). The MMP-9/TIMP-1 axis controls the status of differentiation and function of myelin-forming Schwann cells in nerve regeneration. PLoS One.

[73] Wang Y, Dong Y, Song H, Liu Y, Liu M, Yuan Y (2012). Involvement of gecko SNAP25b in spinal cord regeneration by promoting outgrowth and elongation of neurites. Int J Biochem Cell Biol.

[74] Fan W, Dai Y, Xu H, Zhu X, Cai P, Wang L (2014). Caspase-3 modulates regenerative response after stroke. Stem Cells.

[75] Sheedy FJ (2015). Turning 21: Induction of miR-21 as a Key Switch in the Inflammatory Response. Front Immunol.

[76] Javanmardi S, Aghamaali MR, Abolmaali SS, Mohammadi S, Tamaddon AM (2017). miR-21, An Oncogenic Target miRNA for Cancer Therapy: Molecular Mechanisms and Recent Advancements in Chemo and Radio-resistance. Curr Gene Ther.

[77] Wang ZH, Liu T (2019). MicroRNA21 Meets Neuronal TLR8: Non-canonical Functions of MicroRNA in Neuropathic Pain. Neurosci Bull.

[78] Zhang ZJ, Guo JS, Li SS, Wu XB, Cao DL, Jiang BC (2018). TLR8 and its endogenous ligand miR-21 contribute to neuropathic pain in murine DRG. J Exp Med.

[79] Ning XJ, Lu XH, Luo JC, Chen C, Gao Q, Li ZY (2020). Molecular mechanism of microRNA-21 promoting Schwann cell proliferation and axon regeneration during injured nerve repair. RNA Biol.

[80] Wynder C, Hakimi MA, Epstein JA, Shilatifard A, Shiekhattar R (2005). Recruitment of MLL by HMG-domain protein iBRAF promotes neural differentiation. Nat Cell Biol.

[81] Foehr ED, Raffioni S, Fuji R, Bradshaw RA (1998). FGF signal transduction in PC12 cells: comparison of the responses induced by endogenous and chimeric receptors. Immunol Cell Biol.

[82] Meakin SO, MacDonald JI, Gryz EA, Kubu CJ, Verdi JM (1999). The signaling adapter FRS-2 competes with Shc for binding to the nerve growth factor receptor TrkA. A model for discriminating proliferation and differentiation. J Biol Chem.

[83] Xia L, Zhang Y, Dong T (2016). Inhibition of MicroRNA-221 Alleviates Neuropathic Pain Through Targeting Suppressor of Cytokine Signaling 1. J Mol Neurosci.

[84] Yu B, Zhou S, Wang Y, Qian T, Ding G, Ding F (2012). miR-221 and miR-222 promote Schwann cell proliferation and migration by targeting LASS2 after sciatic nerve injury. J Cell Sci.

[85] Huang ZZ, Wei JY, Ou-Yang HD, Li D, Xu T, Wu SL (2016). mir-500-Mediated GAD67 Downregulation Contributes to Neuropathic Pain. J Neurosci.

[86] Liu Y, Wang L, Lao J, Zhao X (2018). Changes in microRNA expression in the brachial plexus avulsion model of neuropathic pain. Int J Mol Med.

[87] Tramullas M, Frances R, de la Fuente R, Velategui S, Carcelen M, Garcia R (2018). MicroRNA-30c-5p modulates neuropathic pain in rodents. Sci Transl Med.

[88] Wang L, Zhou Y, Chen X, Liu J, Qin X (2022). Long-term iTBS promotes neural structural and functional recovery by enhancing neurogenesis and migration via miR-551b-5p/BDNF/TrkB pathway in a rat model of cerebral ischemia-reperfusion injury. Brain Res Bull.

[89] Penn JW, Grobbelaar AO, Rolfe KJ (2012). The role of the TGF-beta family in wound healing, burns and scarring: a review. Int J Burns Trauma.

[90] Date D, Das R, Narla G, Simon DI, Jain MK, Mahabeleshwar GH (2014). Kruppel-like transcription factor 6 regulates inflammatory macrophage polarization. J Biol Chem.

[91] Kim GD, Das R, Goduni L, McClellan S, Hazlett LD, Mahabeleshwar GH (2016). Kruppel-like Factor 6 Promotes Macrophage-mediated Inflammation by Suppressing B Cell Leukemia/Lymphoma 6 Expression. J Biol Chem.

